# Impact of interventions to improve the quality of peer review of biomedical journals: a systematic review and meta-analysis

**DOI:** 10.1186/s12916-016-0631-5

**Published:** 2016-06-10

**Authors:** Rachel Bruce, Anthony Chauvin, Ludovic Trinquart, Philippe Ravaud, Isabelle Boutron

**Affiliations:** Department of Epidemiology, Mailman School of Public Health, Columbia University, New York, NY USA; INSERM, UMR 1153, Centre of Research in Epidemiology and Statistics Sorbonne Paris Cité – (CRESS), METHODS team, Paris, France; Paris Descartes University, Paris, France; Emergency department, Hôpital Lariboisière, Assistance Publique des Hôpitaux de Paris, University Diderot, Paris, France; Centre d’Épidémiologie Clinique, Hôpital Hôtel-Dieu Escalier A2, 1 er étage, Assistance Publique des Hôpitaux de Paris, 1 Place du Parvis Notre-Dame, Paris, 75004 France

**Keywords:** Peer review process, Peer reviewers, Systematic review, Meta-analysis

## Abstract

**Background:**

The peer review process is a cornerstone of biomedical research. We aimed to evaluate the impact of interventions to improve the quality of peer review for biomedical publications.

**Methods:**

We performed a systematic review and meta-analysis. We searched CENTRAL, MEDLINE (PubMed), Embase, Cochrane Database of Systematic Reviews, and WHO ICTRP databases, for all randomized controlled trials (RCTs) evaluating the impact of interventions to improve the quality of peer review for biomedical publications.

**Results:**

We selected 22 reports of randomized controlled trials, for 25 comparisons evaluating training interventions (n = 5), the addition of a statistical peer reviewer (n = 2), use of a checklist (n = 2), open peer review (i.e., peer reviewers informed that their identity would be revealed; n = 7), blinded peer review (i.e., peer reviewers blinded to author names and affiliation; n = 6) and other interventions to increase the speed of the peer review process (n = 3). Results from only seven RCTs were published since 2004. As compared with the standard peer review process, training did not improve the quality of the peer review report and use of a checklist did not improve the quality of the final manuscript. Adding a statistical peer review improved the quality of the final manuscript (standardized mean difference (SMD), 0.58; 95 % CI, 0.19 to 0.98). Open peer review improved the quality of the peer review report (SMD, 0.14; 95 % CI, 0.05 to 0.24), did not affect the time peer reviewers spent on the peer review (mean difference, 0.18; 95 % CI, –0.06 to 0.43), and decreased the rate of rejection (odds ratio, 0.56; 95 % CI, 0.33 to 0.94). Blinded peer review did not affect the quality of the peer review report or rejection rate. Interventions to increase the speed of the peer review process were too heterogeneous to allow for pooling the results.

**Conclusion:**

Despite the essential role of peer review, only a few interventions have been assessed in randomized controlled trials. Evidence-based peer review needs to be developed in biomedical journals.

**Electronic supplementary material:**

The online version of this article (doi:10.1186/s12916-016-0631-5) contains supplementary material, which is available to authorized users.

## Background

The peer review process is a cornerstone to improve the quality of scientific publications [1–3]. It is used by most scientific journals to inform editors’ decisions and to improve the quality of published reports [4]. Worldwide, peer review costs an estimated £1.9 billion annually and accounts for about one-quarter of the overall costs of scholarly publishing and distribution [5, 6]. Despite this huge investment, primary functions of peer reviewers are poorly defined [7] and the impact and benefit of peer review are increasingly questioned [8–13]. Particularly, studies have shown that peer reviewers were not able to appropriately detect errors [14, 15], improve the completeness of reporting [16], or decrease the distortion of the study results [17–19]. Some interventions have been developed and implemented by some editors to improve the quality of peer review [4, 20–22]. In 2007, Jefferson et al. [23] published a systematic review evaluating the effect of processes in editorial peer review through a search performed in 2005. The authors included prospective and retrospective comparative studies and concluded that little empirical evidence was available to support the use of editorial peer review as a mechanism to improve quality in biomedical publications. To our knowledge, no recent systematic review, including studies published over the last 10 years, has been published.

We aimed to perform a systematic review and a meta-analysis of all randomized controlled trials (RCTs) evaluating the impact of interventions to improve the quality of peer review in biomedical journals.

## Methods

### Search strategy

We searched the Cochrane Central Register of Controlled Trials (CENTRAL; June 2015, Issue 6), MEDLINE (via PubMed), Embase, and the WHO International Clinical Trials Registry Platform for all reports of RCTs evaluating the impact of interventions aiming to improve the quality of peer review in biomedical journals (last search: June 15, 2015). Biomedical research is defined as the area of science devoted to the study of the processes of life, the prevention and treatment of disease, and the genetic and environmental factors related to disease and health. We also searched the Cochrane Database of Systematic Reviews to identify systematic reviews on the peer review process. We had no limitation on language or date of publication. Our search strategy relied on the Cochrane Highly Sensitive Search Strategies [24] and the search term “peer review”. We hand-searched reference lists of reports and reviews dedicated to the peer review process identified during the screening process.

### Study selection

Two researchers (RB, AC) independently screened all citations retrieved by using the Resyweb software. We obtained and independently examined the full-text article for all citations selected for possible inclusion. Any disagreements were discussed with a third researcher until consensus was reached.

We included RCTs, whatever the unit of randomization (manuscript or peer reviewers), evaluating any interventions aimed at improving the quality of peer review for biomedical publications regardless of publication language. We excluded RCTs evaluating the presence and effect of peer reviewer bias on the outcome, such as positive outcome bias. Non-randomized studies were excluded. Duplicate publications of the same study were collated for each unique trial.

### Interventions

We pre-specified the categorization of the interventions evaluated as follows [4]:

1. Training, which included training or mentoring programs for peer reviewers to provide instructional support for appropriately evaluating important components of manuscript submissions. These interventions directly target the ability of peer reviewers to appropriately evaluate the quality of the manuscripts.
2. Addition of peer reviewers for specific tasks or with specific expertise such as adding a statistical peer reviewer, whose main task is to detect the misuse of methods or misreporting of statistical analyses.
3. Peer reviewers’ use of a checklist, such as reporting guideline checklists, to evaluate the quality of the manuscript.
4. “Open” peer review process, whereby peer reviewers are informed that their name would be revealed to the authors, other peer reviewers, and/or the public. The purpose of these approaches is to increase transparency and thus increase the accountability of existing peer reviewers to produce good-quality peer reviews.
5. “Blinded”/masked peer review, whereby peer reviewers are blinded to author names and affiliation. Author names and/or potentially identifying credentials are removed from manuscripts sent for peer review so as to remove or minimize peer reviewer biases that arise from knowledge of and assumptions about author identities.
6. Other interventions. Any other types of interventions identified were secondarily classified.

### Outcomes

The outcomes were as follows:

1. Final manuscript quality (i.e., revised manuscript after all peer review assessments and rounds). Several scales were used to assess manuscript quality. The Manuscript Quality Assessment Instrument, a 34-item scale, each item scored from 1 to 5 [25], aimed to evaluate the quality of the research report (i.e., whether the authors have described their research in enough details and with sufficient clarity so a reader could make an independent judgment about the strength and weakness of their data and conclusion), not the quality of the research itself. This scale is organized along the same dimensions of a journal article (Title and Abstract, Introduction, Methods, Results, Discussion, Conclusions, and General evaluation). Other scales consisted of scales measuring the completeness of reporting [26] based on reporting guideline items (e.g., CONSORT items). A high score indicates a high quality manuscript.
2. Quality of the peer review report, measured by scales such as the Review Quality Instrument [27] or editor routine quality rating scales [28]. A high score indicates a high quality peer review report. Various versions of the Review Quality Instrument exist [27, 29]. The last version of the scale is based on eight items: importance, originality, methodology, presentation, constrictiveness of comments, substantiation of comments and interpretation of results, and a global item. Each item is scored on a 5-point scale. This version had a high level of agreement (K = 0.83) and a good inter-rater reliability (K = 0.31) in the mean total score. No evidence of floor or ceiling effects was found.
3. Rejection rate (i.e., recommendation by peer reviewers about whether or not the reviewed manuscripts should be rejected from publication in the journal).
4. Time spent on the peer review as reported by peer reviewers. The time spent is important to provide an evaluation of the burden and possible cost of the peer review process.
5. Overall duration of the peer review process.

All outcomes were pre-specified except the time spent on the peer review, which was added after inspection of the studies revealed RCTs evaluating interventions to improve the speed of the peer review process.

### Data extraction

Data extraction was performed independently by two researchers who used a standardized data extraction form (Additional file 1). When assessment differed, the item was discussed and consensus was reached. When needed, a third reviewer assessed the report to achieve consensus.

General characteristics of the study recorded included the journal publication, study design, unit of randomization (peer reviewer or manuscripts or both), eligibility criteria for peer reviewers and for manuscripts, number of peer reviewers and/or manuscripts randomized and analyzed, and interventions compared.

The risk of bias within each RCT was assessed by the following domains of the Cochrane Collaboration risk of bias tool [30]: selection bias (methods for random sequence generation and allocation concealment), detection bias (blinding of outcome assessors) and attrition bias (incomplete outcome data). We did not assess performance bias (blinding of participants and providers) because blinding was never possible in this context. Each domain was rated as low, high or unclear risk of bias, according to the guidelines [30]. As recommended by the Cochrane Collaboration, the blinding of outcome assessors and incomplete outcome data domains were assessed at the outcome level. When the risk of bias was unclear, we contacted the authors by email to request more information. When peer reviewers participating in the RCTs were not aware of the study hypothesis or were not aware of the outcome assessed, we considered the detection bias at low risk.

We independently recorded data for each outcome. To avoid selective inclusion of results in systematic reviews, when several results were reported for the selected outcomes (e.g., several scales used to assess the manuscript quality or several time points used to assess the quality of the peer review), we retrieved the results for the outcome reported as the primary outcome in the manuscript or the final or latest time of assessment of the outcome.

For continuous outcomes (i.e., the quality score of the final manuscript, quality of the peer review report, time peer reviewers spent on the peer review), we recorded the mean and standard deviation per arm. If data were not provided in the form of a mean and standard deviation, we derived these from the reported data and produced statistics following the Cochrane handbook recommendations [31].

For dichotomous outcomes (i.e., rejection rate), we recorded the number of manuscripts evaluated and the number of manuscripts for which peer reviewers recommended rejection.

For studies with multiple intervention groups (e.g., blinded peer review and open peer review), we followed the Cochrane recommendations [31] and, when appropriate, we combined all relevant intervention groups for the study into a single group, combined all relevant control intervention groups into a single control group, or selected one pair of interventions and excluded the others.

When required, we extracted the outcome data from the published figures by using Digitizelt [32]. We contacted authors when key data were missing.

Publication bias and small study effect were assessed by visual inspection of funnel plots when appropriate.

### Data synthesis

Treatment effect measures were odds ratios (ORs) for binary outcomes (rejection rate) and standardized mean differences (SMDs) for continuous outcomes (quality score of the final manuscript, quality of peer review report, and mean difference for the time peer reviewers spent on the peer review). RCTs measured the quality of the manuscript and the peer review in various ways (using different scoring systems), so we measured the intervention effects by using SMDs.

Because of the diversity of the interventions and settings, we used random-effects meta-analysis (Dersimonian and Laird model). We assessed heterogeneity by visual inspection of the forest plots, Cochrane’s homogeneity test, and the I^2^ statistic [33]. In the meta-analysis of RCTs assessing open peer review, we performed one subgroup analysis to evaluate the impact of different intensities of open peer review (open to other peer reviewers/open to authors/publicly available). Analyses involved use of Review Manager 5.3 (Cochrane Collaboration).

RCTs assessing training interventions reported a mixture of change from baseline and final value scores regarding the quality of the peer review report. As recommended, we did not combine the final value and change scores together as SMDs [34].

## Results

### Study selection and characteristics of included studies

**Fig. 1:**
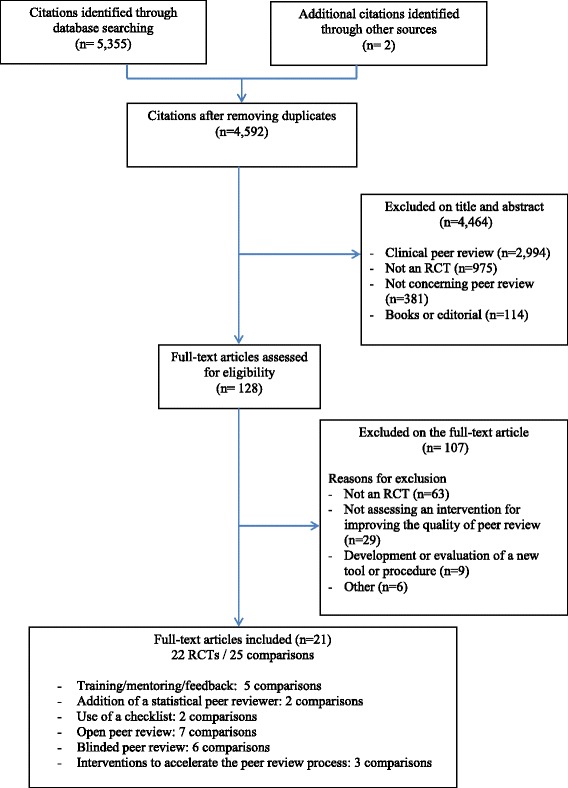
Study selection flow diagram

Figure 1 provides the flow diagram for the study selection. From the 4592 citations retrieved, we selected 21 published articles reporting 22 RCTs and 25 comparisons, evaluating training interventions (n = 5), the addition of a statistical peer reviewer (n = 2), use of a checklist (n = 2), open peer review (n = 7), blinded peer review (n = 6), and other interventions to increase the speed of the peer review process (n = 3). Only seven RCTs were published since 2004. Searching the WHO trial register platform did not identify any RCT.

The characteristics of the selected studies are presented in Table 1. The description of the interventions assessed in each RCT is provided in Additional file 2.

**Table 1:** General characteristics of the trials included

Author, Year, Journal publishing the study	Intervention/Comparator	Journal(s) involved in the study	Peer reviewers (n)	Manuscripts (n)/Peer review reports (n)	Unit of randomization	Outcomes (Scale)	Sample size (randomized/analyzed)	Risk of bias
Training/Mentoring/Feedback								
Callaham, JAMA 2002 Study 1a [36]	Feedback by editors/usual process	Annals of Emergency Medicine	Low volume low quality^a^ peer reviewers (n = 51)	Manuscripts submitted to the journal (n = NR)/182 peer review reports	Peer reviewers	The quality of peer review report (using editor routine quality scale)	51 randomized/35 analyzed	- Rs: Low - Al: Low - D: Low - At: High
Callaham, JAMA 2002 Study 1b [36]	Feedback by editors/usual process	Annals of Emergency Medicine	Low volume, average quality^b^ peer reviewers (n = 127)	Manuscripts submitted to the journal (n = NR)/324 peer review reports	Peer reviewers	The quality of peer review report (using editor routine quality scale)	127 randomized/95 analyzed	- Rs: Low - Al: Low - D: Low - At: High
Callaham, 2002 Ann Emerg Med Study 2 [37]	Feedback by editors/usual process	Annals of Emergency Medicine	Average quality^b^ peer reviewers (n = 150)	Manuscripts submitted to the journal (n = NR)/79 peer review reports	Peer reviewers	The quality of peer review report (using editor routine quality scale)	150 randomized/22 analyzed	- Rs: Low - Al: Low - D: Low - At: High
Houry, 2012 BMC Med Educ [38]	Training (workshop)/usual process	Annals of Emergency Medicine	New peer reviewers (n = 50)	Manuscripts submitted to the journal (n = 490)/490 peer review reports	Peer reviewers	The quality of peer review report (using editor routine quality scale)	50 randomized/46 analyzed	- Rs: Low - Al: Unclear - D: Unclear - At: Low
Schroter, 2004 BMJ [35]	Training (face-to-face or self-training)/usual process (The two intervention groups, face-to-face and self-training, were pooled in the meta-analysis)	British Medical Journal	Consenting peer reviewers (n = 609)	One fabricated manuscript with errors (n = 1)^c^/418 peer review reports	Peer reviewers	1) The quality of peer review report (using the RQI Version 3.2) 2) The rejection rate 3) The time spent on the review	609 randomized/418 analyzed	- Rs: Low - Al: Low - D: Low - At: High
Statistical peer review								
Arnau, 2003 Med Clin (Barc) [39]	Adding a statistical peer reviewer/usual process	Medicina Clinica	Statistical peer reviewers (n = NR)	Manuscripts submitted to the journal (n = 82)	Manuscripts	The final manuscript quality (using the MQAI)	82 randomized/43 analyzed	- Rs: Low - Al: Low - D: Low - At: High
Cobo, 2007 PLOS One [40]	2 × 2 factorial design comparing/adding a statistical peer reviewer/use of a reporting guidelines checklist/both/usual process (we selected only the two groups adding a statistical peer reviewer/usual process in the analysis)^d^	Medicina Clinica	Statistical peer reviewers (n = 39)	Consecutive manuscripts submitted to the journal (n = 68)	Manuscripts	The final manuscript quality (using the MQAI)	68 randomized/62 analyzed	- Rs: Low - Al: Low - D: Low - At: Low
Checklist								
Cobo, 2007 PLOS One [40]	2 × 2 factorial design comparing adding a statistical peer reviewer/use of a reporting guidelines checklist/both/usual process (we selected only the two groups use of a reporting guidelines checklist/usual process in the analysis)^d^	Medicina Clinica	Statistical peer reviewers (n = 39)	Consecutive manuscripts submitted to the journal (n = 69)	Manuscripts	The final manuscript quality (using the MQAI)	69 randomized/60 analyzed	- Rs: Low - Al: Low - D: Low - At: Low
Cobo, 2011 BMJ [41]	Use of a reporting guidelines checklist/usual process	Medicina Clinica	A senior statistician (n = 1)	Consecutive manuscripts submitted to the journal (n = 92)	Manuscripts	The final manuscript quality (using the MQAI)	92 randomized/92 analyzed	- Rs: Low - Al: Low - D: Low - At: Low
Open peer review (i.e., identity of peer reviewers revealed to the authors, other peer reviewers, and/or general public)								
Das Sinha, 1999 Natl Med J India [42]	Pairs of reviewers were identified to assess each manuscript and the two reviewers were randomized; one to be informed they would have their identity revealed to the other peer reviewer and one to remain anonymous	The National Medical Journal of India	Peer reviewers of the journal (n = 156, 78 pairs)	Manuscripts submitted to the journal (n = 100)/156 peer review reports	Pairs of peer reviewers	1) The quality of peer review report (using editor routine quality scale) 2) The rejection rate	100 randomized/78 analyzed	- Rs: Low - Al: Low - D: Low - At: Low
Godlee, 1998 JAMA [47]	Five groups: 1) ask to sign their report + blinded to authors name and affiliation; 2) ask to remain anonymous + blinded to authors name and affiliation; 3) ask to sign their report + unblinded to authors name and affiliation; 4) ask to remain anonymous + unblinded to authors name and affiliation/usual process We pooled groups 1 and 3 vs. 2 and 4 A fifth group where peer reviewers were unaware of the study for which the manuscript was sent according to the usual process was not taken into account in the analysis	British Medical Journal	Peer reviewers of the journal (n = 420)	One fabricated manuscript with errors (n = 1)/184 peer review reports	Peer reviewers	The rejection rate	360 randomized/184 analyzed	- Rs: Low - Al: Low - D: Low - At: High
Van Rooyen, 1998 JAMA [48]	Open to peer reviewers (combination of blinded and unblinded to authors identity) vs. anonymous (combination of blinded and unblinded to authors identity)	British Medical Journal	Peer reviewers of the journal (n = NR)	Consecutive manuscripts submitted to the journal (n = 527)	Manuscripts & peer reviewers	1) The quality of peer review report (using the RQI Version 3.2) 2) The time spent on the review	527 manuscript randomized/598 reviews analyzed	- Rs: Low - Al: Unclear - D: Low - At: Unclear
Van Rooyen, 1999 BMJ [43]	Identity revealed to authors/peer reviewers remained anonymous to authors For each manuscript, a pair of reviewers were identified and each reviewer was randomized to have their identity revealed to authors or remain anonymous	British Medical Journal	Peer reviewers of the journal (n = 250)	Consecutive manuscripts submitted to the journal (n = 125)/113 manuscripts assessed/226 peer review reports	Peer reviewers	1) The quality of peer review report (using the RQI Version 4) 2) The rejection rate 3) The time spent on the review	250 randomized/226 analyzed	- Rs: Low - Al: Unclear - D: Low - At: High
Van Rooyen, 2010 BMJ [44]	Identity revealed to general public/peer reviewers signed their review for authors and other peer reviewers	British Medical Journal	Peer reviewers of the journal (n = 471)	Consecutive manuscripts submitted to the journal (n = 558)	Manuscripts	1) The quality of peer review report (using the RQI Version 4) 2) The rejection rate 3) The time spent on the review	558 manuscript randomized/471 analyzed	- Rs: Unclear - Al: Unclear - D: Low - At: Low
Vinther, 2012 Dan Med [45]	Pairs of reviewers were identified to assess each manuscript; for each manuscript, peer reviewers were randomized to have their identity revealed to authors/remained anonymous to authors	The Journal of Danish Medical Association	Peer reviewers of the journal (n = 380)	Manuscripts submitted to the journal (n = 190)/364 peer review reports	Peer reviewers	1) The quality of peer review report (using the RQI Version 4) 2) The rejection rate	380 randomized/364 analyzed	- Rs: Unclear - Al: Unclear - D: Low - At: Low
Walsh, 2000 Br J Psychiatry [46]	Identity revealed to authors/peer reviewers remained anonymous to authors	British Journal of Psychiatry	Peer reviewers of the journal (n = 245)	Manuscripts submitted to the journal (n = 408)/354 peer review reports	Manuscript	1) The quality of peer review report (using the RQI Version 3.2) 2) The rejection rate 3) The time spent on the review	408 manuscripts randomized/354 analyzed	- Rs: Low - Al: Unclear - D: Low - At: High
Blinded peer review (i.e., peer reviewers are blinded of the authors name and affiliation)								
Alam, 2011 Br J Dermatol [49]	Randomization of four peer reviewers for each manuscript, two randomized to assess a blinded version of the manuscript, two to assess an unblinded version of the manuscript	Dermatologic Surgery	Volunteer peer reviewers of the journal (n = 20)	Consecutive manuscripts submitted to the journal (n = 40)/160 peer review reports	Peer reviewers & manuscript	The rejection rate	20 peer reviewers/40 manuscripts 160 peer review reports analyzed 6 peer reviewers failed to do their review and were replaced (3 blinded/3 unblinded)	- Rs: Unclear - Al: Unclear - D: Low - At: Low
Fisher, 1994 JAMA [50]	Identification of four peer reviewers for each manuscript, two randomized to assess a blinded version of the manuscript, two to assess an unblinded version of the manuscript	Journal of Developmental and Behavioral Pediatrics	Peer reviewers of the journal (n = 228)	Consecutive manuscripts submitted to the journal (n = 57)/228 peer review reports	Peer reviewers	The rejection rate	228 randomized/220 analyzed	- Rs: Low - Al: Unclear - D: Low - At: Low
Godlee, 1998 JAMA [47]	Five groups: 1) ask to sign their report + blinded to authors name and affiliation; 2) ask to remain anonymous + blinded to authors name and affiliation; 3) ask to sign their report + unblinded to authors name and affiliation; 4) ask to remain anonymous + unblinded to authors name and affiliation/usual process We pooled groups 1 and 2 vs. 3 and 4 A fifth group where peer reviewers were unaware of the study for which the manuscript was sent according to the usual process was not taken into account in the analysis	British Medical Journal	Peer reviewers of the journal (n = 360)	One fabricated manuscript with errors (n = 1)/130 peer review reports	Peer reviewers	The rejection rate	240 randomized/130 analyzed	- Rs: Low - Al: Low - D: Low - At: High
Justice, 1998 JAMA [51]	Identification of two peer reviewers for each manuscript, one randomized to assess a blinded version of the manuscript, one to assess an unblinded version of the manuscript	Annals of Emergency Medicine, Annals of Internal Medicine, JAMA, Obstetrics & Gynecology and Ophthalmology	Peer reviewers of journals (n = NR)	Manuscripts submitted to journals (n = 92)/77 manuscripts with two peer review reports	Peer reviewers	The quality of peer review report (using editor routine quality scale)	92 manuscript with two reviewers/77 manuscript with two reviewers reports analyzed	- Rs: Low - Al: Unclear - D: Low - At: Unclear
McNutt, 1990 JAMA [52]	Identification of two peer reviewers for each manuscript, one randomized to assess a blinded version of the manuscript, one to assess an unblinded version of the manuscript	Journal of General Internal Medicine	Peer reviewers of the journal (n = NR)	Manuscripts submitted to the journal (n = 127)/252 peer review reports	Peer reviewers	1) The quality of peer review report (using editor routine quality scale) 2) The time spent on the review	127 manuscript with two reviewers/127 and 125 reviewers reports analyzed	- Rs: Low - Al: Unclear - D: Low - At: Low
Van Rooyen, 1998 JAMA [48]	Identification of two peer reviewers for each manuscript, one randomized to assess a blinded version of the manuscript, one to assess an unblinded version of the manuscript This study also assessed masked versus unmasked review (See above in open peer review intervention)	British Medical Journal	Peer reviewers of the journal (n = NR)	Consecutive manuscripts submitted to the journal included in this study (n = 309)	Peer reviewers & manuscripts	1) The quality of peer review report (using the RQI Version 3.2) 2) The time spent on the review	309 manuscripts with two reviewers randomized/618 peer reviews reports analyzed	- Rs: Low - Al: Unclear - D: Low - At: Unclear
Accelerate the peer review process								
Johnston, 2007 Ann Neurol [54]	Early screening by editors/formal external review	Annals of Neurology	Peer reviewers of the journal (n = 386)	Consecutive manuscripts submitted to the journal (n = 351)	Manuscripts	The time to a manuscript decision	351 manuscripts randomized/351 reviews analyzed	- Rs: Unclear - Al: Low - D: low - At: Low
Neuhauser, 1989 Medical Care [55]	Calling first peer reviewers/sending out manuscript without a prior phone call	Medical Care	Peer reviewers of the journal (n = NR)	Manuscripts submitted to the journal (n = 95)	Peer reviewers	The overall time for the peer review process	95 manuscripts with two peer reviewers randomized	- Rs: Unclear - Al: Unclear - D: Low - At: Low
Pitkin, 2002 JAMA [53]	Asking first: referees received information about manuscript by fax and indicated their willingness to review/editors mailed the manuscript and asked to return the review	Obstetrics & Gynecology	Peer reviewers of the journal (n = NR)	Consecutive manuscripts submitted to the journal (n = 283) Identification of two peer reviewers for each manuscript	Peer reviewers	1) The overall time for the peer review process 2) The quality of peer review report (using editor routine quality scale)	283 manuscripts with two reviewers randomized and analyzed	- Rs: Low - Al: Low - D: Low - At: Low

Rs, Random sequence generation; Al, Allocation concealment; D, Detection bias (blinding of outcome assessment); At, Attrition bias (incomplete outcome data); NR, not reported. The Manuscript Quality Assessment Instrument (MQAI) is a 34-item scale, each item scored from 1 to 5, aimed to evaluate the quality of the research report. The Review Quality Instrument (RQI) is an 8-item scale, each item scored from 1 to 5, aimed to evaluate the quality of the peer review report

^a^Reviewers with a median score of 3 or lower on a quality scale of 1 to 5 routinely used by editors for the reviews they performed in the previous 2 years

^b^Reviewers with a median score of 4 or lower on a quality scale of 1 to 5 routinely used by editors for the reviews they performed in the previous 2 years

^c^Peer reviewers provided three peer review reports for three different manuscripts; we selected the assessment of the last manuscript

^d^We did not consider the group: “clinical reviewer + statistician + checklist reviewer”, because we were interested in the effect of the use of a checklist or adding a statistician reviewer, not the effect of both

The 22 RCTs were published in 12 different journals. The study design varied according to the interventions evaluated and consisted of randomizing peer reviewers (n = 15), manuscripts (n = 6) or both (n = 4). The manuscripts used in the RCTs were submitted by authors to the system by the usual process (n = 20) or were fictitious manuscripts specifically prepared for the study with error added (n = 2).

### Data synthesis

#### Training

**Fig. 2:**
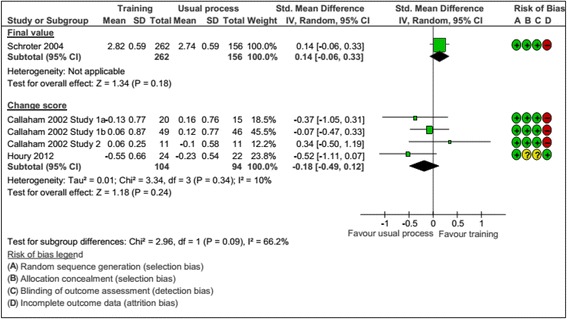
Impact of training versus usual process: standardized mean difference (SMD) of the quality of the peer review report

Four RCTs, involving five comparisons and 616 peer reviewers, compared the impact of training interventions to the standard peer review procedure for the given journal on the quality of the peer review report [35–38]. Four comparisons of the *Annals of Emergency Medicine* [36–38] evaluated feedback (n = 2), training (n = 1) and mentoring (n = 1), and one comparison of the *British Medical Journal* evaluated training [35]. Different kinds of training were evaluated: structured workshops with journal editors or self-taught training with a package created especially for peer reviewers. Mentoring consisted of new peer reviewers discussing their review with a senior peer reviewer before sending the review. Feedback consisted of peer reviewers receiving a copy of the editor’s rating of their review. One study evaluated two kinds of interventions: self-taught or face-to-face [35]. For this study, we pooled these two arms versus the usual process. The risk of attrition bias was high for four RCTs (Additional file 3). Training had no impact on the quality of the peer review report, but the heterogeneity across studies was high (66.2 %) and the 95 % confidence interval (95 % CI) relatively large (Fig. 2). Only one study assessed the time to perform the peer review and the rejection rate with training [35]: training did not affect the time to review manuscripts (mean (SD), 116.9 (69.4) min for training vs. 108.5 (70.5) min for the usual process; *P* = 0.38) – but did increase recommendations to reject manuscripts (OR, 0.47; 95 % CI, 0.42 to 0.78).

#### Addition of a statistical peer reviewer

**Fig. 3:**
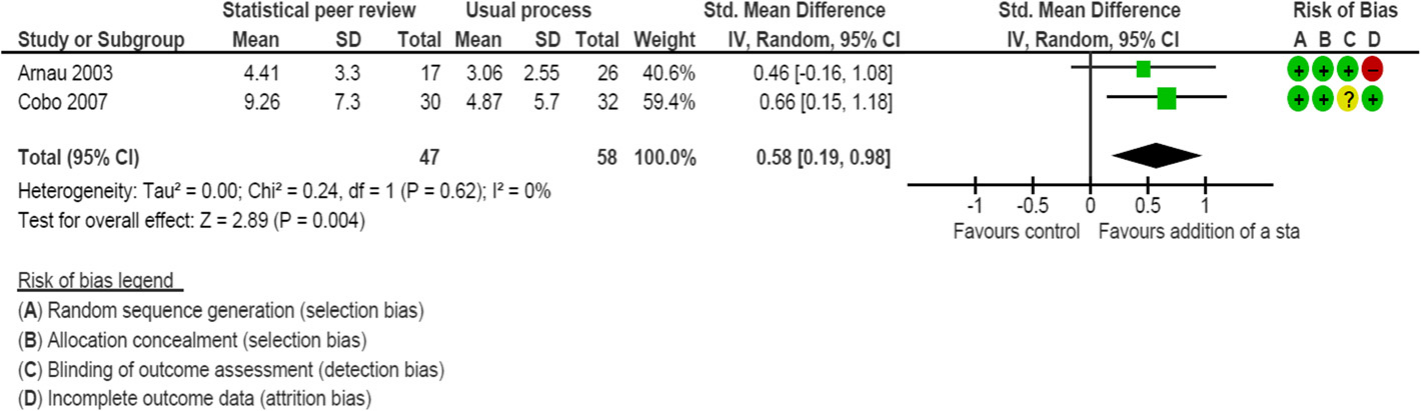
Impact of adding a statistical peer review versus usual process: standardized mean difference (SMD) of the final manuscript quality

Two RCTs examined the impact of adding a statistical peer reviewer on the final manuscript quality [39, 40]. All RCTs involved the *Medicina Clínica* journal (a Spanish journal of internal medicine) and were conducted by the same research team. The RCT by Cobo et al. [40] was a 2 × 2 factorial design comparing the addition of a statistical peer reviewer, the use of a checklist, both interventions, and the usual process. For this analysis, we used the arms dedicated to the usual process and the addition of a statistical peer reviewer. The risk of bias was low for these two studies (Additional file 3). Overall, 105 manuscripts were included in the meta-analysis. Adding a statistical peer reviewer increased the quality of the final manuscript as compared to the usual process (combined SMD, 0.58; 95 % CI, 0.19 to 0.98; I^2^ = 0 %; Fig. 3).

#### Use of a reporting guideline checklist

**Fig. 4:**
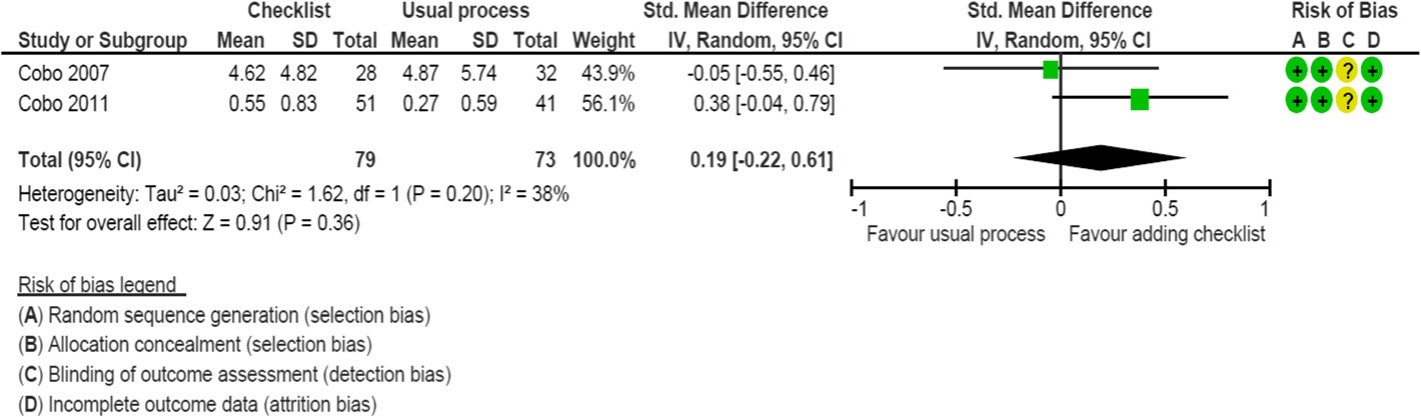
Impact of using checklist versus usual process: standardized mean difference (SMD) of the final manuscript quality

Two studies evaluated the impact of peer reviewers using a reporting guideline checklist on the quality of the final manuscript [40, 41]. These studies were performed by the same research team and involved the *Medicina Clínica* journal. For the RCT by Cobo [40], we used the arms dedicated to the usual process and the addition of a checklist. The risk of bias was low (Additional file 3). Overall, 152 manuscripts were included in the meta-analysis. Overall, use of a checklist had no effect on the quality of the final manuscript as compared with the usual process (combined SMD, 0.19; 95 % CI, –0.22 to 0.61; I^2^ = 38 %; Fig. 4).

#### “Open” peer review

**Fig. 5:**
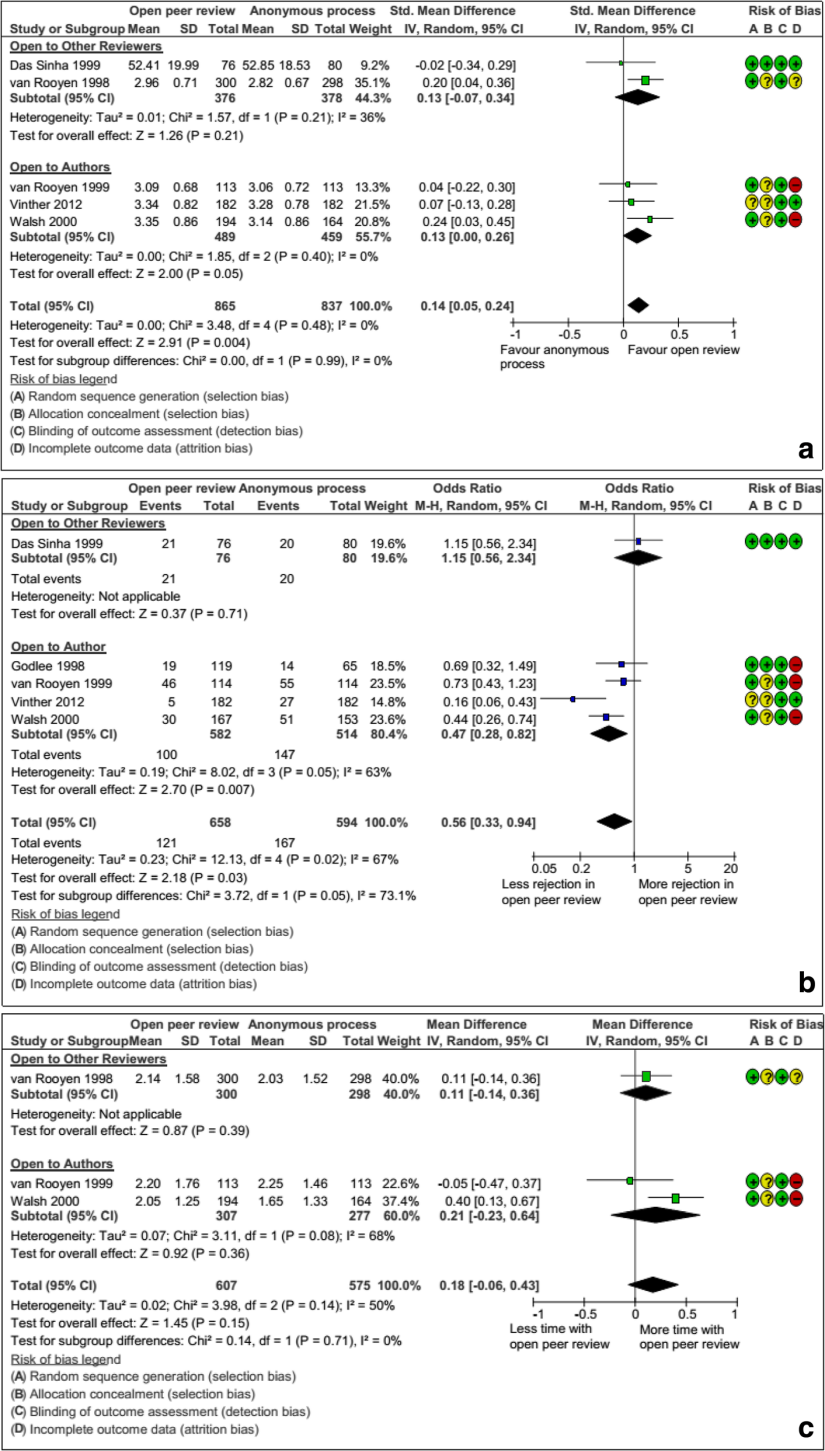
**a** Impact of the “open” review interventions versus anonymous process (anonymous to reviewers, authors or public): standardized mean difference (SMD) of the quality of the peer review report. **b** Impact of the “open” review interventions versus anonymous process (anonymous to reviewers, authors or public): odds ratio (OR) of peer reviewers’ recommendation for rejection. **c** Impact of the “open” review interventions versus anonymous process (anonymous to reviewers, authors or public): standardized mean difference (SMD) of the time peer reviewers spent on the peer review

Seven RCTs assessed the impact of open review interventions [42–48], consisting of informing peer reviewers that their identity would be revealed to other peer reviewers only (n = 2), to the authors of the manuscript being reviewed (n = 3), and to the general public via web-posting (n = 1). One study evaluated both open peer review and peer reviewers blinded or not to the authors’ identity [47]. One study was not included in the meta-analysis because the usual process was not the same comparator as for other RCTs [44]. In fact, authors compared signed peer reviews posted on the web versus signed peer reviews for authors and other peer reviewers. A total of 1702 peer reviewers, 1252 manuscripts, and 1182 manuscripts were included in the meta-analysis of the quality of peer review reports, the rejection rate, and the time peer reviewers spent on peer review, respectively. With open peer review, the quality of the peer review report increased (combined SMD, 0.14; 95 % CI, 0.05 to 0.24, I^2^ = 0 %; Fig. 5a) and the recommendation to reject manuscripts for publication decreased, but the heterogeneity across studies was high (OR, 0.56; 95 % CI, 0.33 to 0.94; I^2^ = 67 %; Fig. 5b). Subgroup analysis by type of open peer review (i.e., open to other peer reviewers or to authors) did not show evidence of difference. Three of the included studies evaluated the time peer reviewers spent on the peer review for each manuscript (Fig. 5c). Overall, the time peer reviewers spent on the peer review did not differ between the open peer-review and standard peer review groups (combined MD, 0.18; 95 % CI, –0.06 to 0.43, I^2^ = 50 %). Subgroup analysis by type of open peer review (i.e., open to other peer reviewers or open to authors) did not show evidence of difference.

#### Blinded peer review

**Fig. 6:**
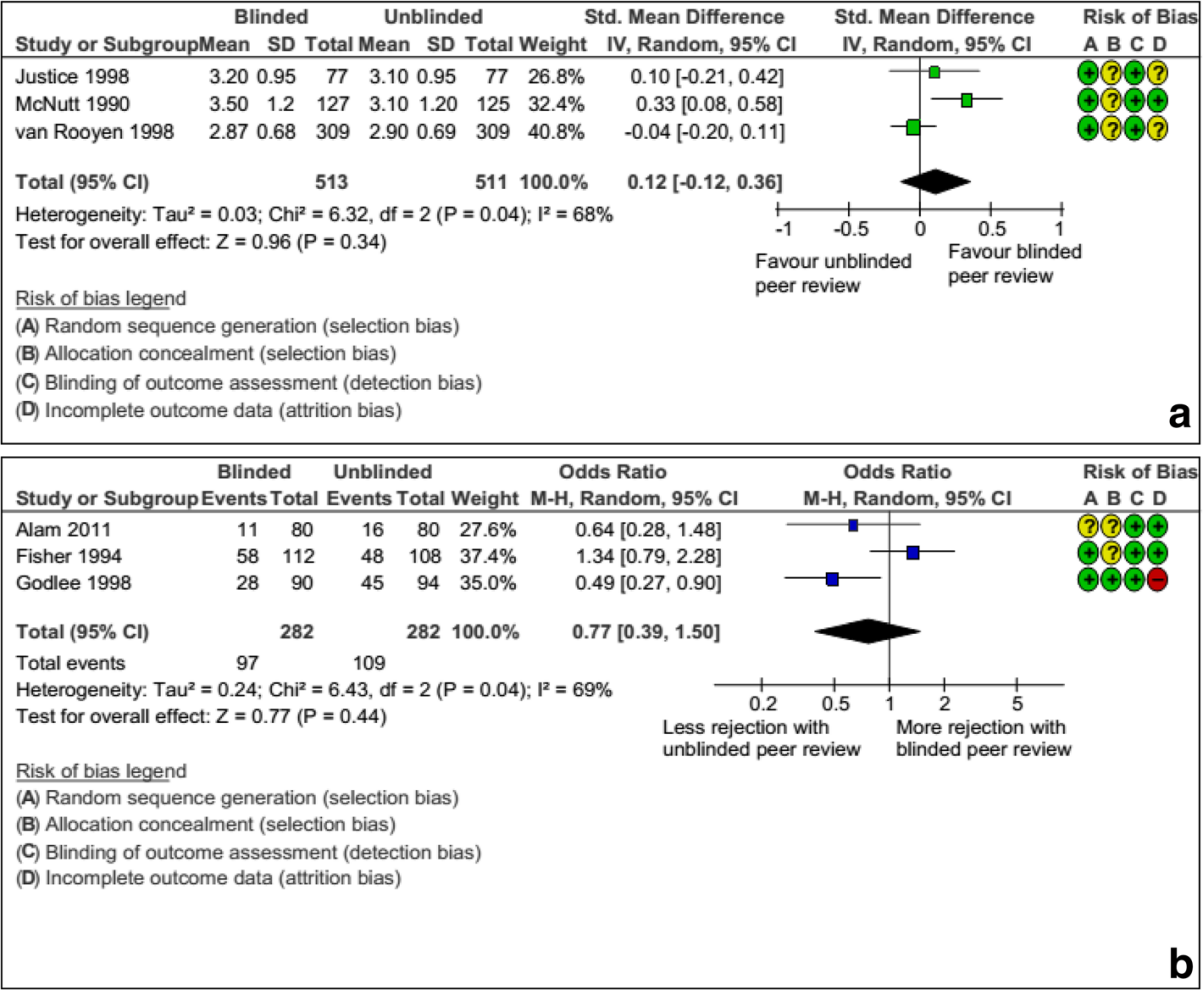
**a** Impact of blinded peer review interventions versus usual process: standardized mean difference (SMD) of the quality of the peer review report. **b** Impact of blinded peer review interventions versus usual process: odds ratio (OR) of peer reviewers’ recommendation for rejection

Among six RCTs evaluating blinded peer review, three evaluated the impact on the quality of the peer review report and three the rejection rate [47–52]; 1024 and 564 manuscripts, respectively, were included in the meta-analyses. Blinded peer review did not affect the quality of the peer review report (SMD, 0.12; 95 % CI, –0.12 to 0.36, I^2^ = 68 %) or the rate of rejection (OR, 0.77; 95 % CI, 0.39 to 1.50, I^2^ = 69 %), but the confidence interval was large and the heterogeneity among studies substantial (Fig. 6a and b).

#### Interventions to accelerate the peer review process

For three RCTs evaluating interventions to improve the duration of the peer review process [53–55], the interventions consisted of first asking the peer reviewers whether they agreed to review the manuscript versus directly sending the manuscript to review [53] or calling peer reviewers versus sending the manuscript [55]. Finally, the mean duration of the peer review process was decreased with an intervention based on early screening of manuscripts by editors versus the usual process [54]. We could not perform a meta-analysis because outcomes were incompletely reported.

## Discussion

Despite the essential role of peer review in the biomedical research enterprise, our systematic review identified only 22 RCTs evaluating interventions to improve the peer review quality; results of only seven RCTs were published over the past 10 years. To our knowledge, no ongoing trial is registered at trial registries. Our results provide little evidence of an effect of training of peer reviewers and the use of a checklist on the quality of the peer review report and the final manuscript. However, the amount of evidence is scarce and the methodological quality raises some concerns. Only two RCTs evaluated the impact of adding a peer reviewer with statistical expertise and showed favorable results on the quality of the final manuscript. However, these studies were performed in a single journal and need to be reproduced in other contexts. Open peer review, routinely implemented by several journals such as the *British Medical Journal* or BioMed Central journals, had a small favorable impact on the quality of the peer review report, increased the time peer reviewers spent on the review, and decreased recommendations to reject manuscripts. Blinded peer review i.e. peer reviewers are blinded to author names and affiliation did not affect the rate of rejection. The goal of blinding peer review is not to change the quality of the review but to favor objective and fair review. However, evaluating whether a review is fair and objective is a difficult or even impossible task. This explains why researchers focused on the quality of the report and the rejection rate. Nevertheless, the lack of difference observed in these outcomes should not be interpreted as a lack of usefulness of blinding. Finally, interventions to improve the speed of the process were heterogeneous.

The first International Congress on Peer Review in Biomedical Publication was held in Chicago in 1989 [56], and aimed to stimulate research on peer review. In 2007, Jefferson et al. [23] published a Cochrane systematic review on editorial peer review and concluded that a large, well-funded program of research on the effects of editorial peer review was urgently needed. Several years later, despite some initiatives such as the European COST action PEERE [57], research involving experimental design is scarce in this field, which is alarming considering the cost and central role of this process in biomedical science.

The methodological problems in studying peer review are many and complex. First, the primary functions of peer review are poorly defined. According to the editor, the role of peer reviewer varies from assessing the novelty of the study results, to assessing the scientific rigor, to assessing the clinical relevance, etc. [7]. The choice of the design is also difficult. In our review, the study designs involved the randomization of peer reviewers to assess a sample of manuscripts submitted to a journal or fabricated manuscripts developed for the RCT or the randomization of manuscripts to be assessed by a sample of peer reviewers. The selection of journals, peer reviewers and manuscripts will affect the applicability of the study results. Most RCTs were performed in a single journal and limited the generalizability of results to very specific contexts. Journal culture, standard review procedures, sub-specialties, and the experience of peer reviewers all play a role in the changed quality of reviews observed. For example, statistical peer review was studied in only one publication (*Medicina Clínica*), in three RCTs conducted by the same study team; although the results are reliable, the external validity is limited.

Bias related to the lack of blinding is also problematic when designing these RCTs. Concealing study aims and procedures from study subjects is difficult and even impossible for some of these interventions. For instance, for RCTs of participants randomized to be blinded to author identity in blinding interventions, unintentional discovery or speculation of the author’s identity was reported. Similarly, due to controversial opinions of open peer review, some of the studies for open peer-review interventions were subject to bias because of a large number of potential subjects declining to participate or not completing the allocated peer review.

The choice of relevant outcomes is also difficult in this domain. The outcomes assessed were mainly the editor’s subjective assessment or use of validated scales to assess the quality of the peer review report [35–38] or the final manuscript [39–41]. However, the choice of the criteria to consider the appropriateness of a peer review report can be debatable and can vary according to the editor in charge. For example, a study showed a discrepancy between editors and peer reviewers regarding items considered most important when assessing the report of an RCT [58]. Further, the validity of some of these outcomes (e.g., editors’ routine scales) and the minimal editorial relevant difference could be questioned. Likewise, the association between recommendations to reject and the quality of the peer review is not well understood, and the proposed goal of these interventions in terms of rejection rate is not well defined. A high rejection rate could be related to the quality of the manuscript but also to the interest of the topic or to peer reviewer subjectivity. In our review, no studies evaluated the association between a recommendation of rejection and the quality of the published study. Finally, the quality of the final manuscript is not solely the results of the peer reviewers’ assessment. In fact, some relevant peer reviewer suggestions might not have been acted upon, either because the authors refused or the editor did not enforce them. Of note, articles included in our systematic review evaluated the final manuscript quality after a single round of peer review. Further, for some journals, copy editing will also impact the article quality.

Our systematic review has some limitations. We selected only RCTs performed in the field of biomedical research and we cannot exclude that some interventions were developed and evaluated in other fields. We did not search some more specific bibliographic databases such as Cumulative Index to Nursing and Allied Health Literature (CINAHL). However, our search was extensive and performed according to Cochrane standards. The limited number of RCTs identified, their small sample sizes, their methodological quality, and their applicability limit the interpretation of our results. Finally, reporting bias can be a concern in systematic reviews and because of the limited number of RCTs, we could not explore reporting bias. However, in this particular area, both negative and positive results are of interest to the peer-reviewed academic journal community. It is unlikely that additional RCTs meeting inclusion criteria were unreported or that results from the RCTs were selectively omitted. Still, the limitations of this body of evidence prohibit our ability to make definitive general recommendations regarding their implementation in peer review editorial processes for biomedical publications.

## Conclusions

Currently, we cannot provide conclusive recommendations on the use of interventions to improve quality of peer review from this body of evidence and its limitations. Since the publication of a previous systematic review on the topic [23], even with the inclusion of a number of more recent RCTs, the state of the evidence falls short of generating empirical support. Editorial boards and decision-makers of the publication process structure should remain aware of the uncertainty in these intervention methods and weigh the shortcomings and practical challenges before implementing them. These results highlight the urgent need to clarify the goal of the peer review process, the definition of a good quality peer review report, and the outcomes that should be used. In the longer term, these results reiterate the need for additional experimentation on this topic and exploration into the drivers of publication quality. In fact, the evidence base for recommendations needs to be strengthened. Further research is needed to identify interventions that could improve the process and assess those interventions in well-performed trials.

## Abbreviations

MD, mean difference; OR, odds ratio; RCT, randomized controlled trials; SMD, standardized mean difference.

## Additional files

Additional file 1: Appendix 1. Data extraction and assessment form. (DOC 128 kb) Additional file 2: Appendix 2. General characteristics of the RCTs selected. (DOC 94 kb) Additional file 3: Appendix 3. Risk of bias summary: the review authors’ judgments about each risk of bias item for each included study. (DOC 278 kb)
