# Supplementary material for: Impact of interventions to improve the quality of peer review of biomedical journals: a systematic review and meta-analysis
Source: BMC Med. 2016 Jun 10;14:85. doi: 10.1186/s12916-016-0631-5 (PMC4902984; doi:10.1186/s12916-016-0631-5)
Supplement: Additional file 1: Appendix 1. — Data extraction and assessment form. (DOC 128 kb) [file 12916_2016_631_MOESM1_ESM.doc]

###### Appendix 1. Data Extraction & Assessment Form

| **Study ID:** | | | Data extractor: □ RB □ AC | |
| --- | --- | --- | --- | --- |
| First Author: | | Year of study publication: | | |
| Title:  Publication: | | | | |
| Registered? □ yes □ no | Registry: | | | Registry #: |

1. General Information:

| Extracted from | | □ full-text article □ abstract □ protocol □ other (specify____________________) | | |
| --- | --- | --- | --- | --- |
| Language | | □ English □ other (specify____________________) | | |
| Funding source of study | | □ public □ private □ both □ none specified □ unclear | | |
| □ monocenter □ multicenter (specify # of centers________) □ not reported  Countries:  Center type: □ journal □ medical school □ residency program □ other (specify____________________) | | | | |
| Trial design | □ 2-arm RCT □ cluster RCT □ other (specify____________________) | | | |
| Recruitment period |  | | Randomized  *(total #, rate)* |  |
| Study completion  *(total #, rate)* |  | | Intervention adherence  *(total #, rate)* |  |

2. Participants:

|  | **Control** | **Intervention Arm (1)** | **Intervention Arm (2)** |
| --- | --- | --- | --- |
| Age *(mean, range, SD)* |  |  |  |
| Sex *(male:female ratio)* |  |  |  |
| Country |  |  |  |
| Academic rank |  |  |  |
| Editorial board position | □ yes □ no  □ unclear | □ yes □ no  □ unclear | □ yes □ no  □ unclear |
| Grant reviewer | □ yes □ no  □ unclear | □ yes □ no  □ unclear | □ yes □ no  □ unclear |
| IRB reviewer | □ yes □ no  □ unclear | □ yes □ no  □ unclear | □ yes □ no  □ unclear |
| Years in research  *(mean, range, SD)* |  |  |  |
| Area(s) of expertise |  |  |  |
| Journal type & impact level *(if applicable)* |  |  |  |

3. Intervention(s):

| **Control** | **Control** | **Intervention (1)** | **Intervention (2)** |
| --- | --- | --- | --- |
| Supervision | □ supervised  □ unsupervised  □ both □ unclear | □ supervised  □ unsupervised  □ both □ unclear | □ supervised  □ unsupervised  □ both □ unclear |
| Teaching style | □ passive □ hands-on  □ both □ unclear | □ passive □ hands-on  □ both □ unclear | □ passive □ hands-on  □ both □ unclear |
| Mode of teaching | □ face-to-face  □ online  □ print  □ combination  □ unclear | □ face-to-face  □ online  □ print  □ combination  □ unclear | □ face-to-face  □ online  □ print  □ combination  □ unclear |
| Frequency |  |  |  |
| Duration |  |  |  |
| Intensity *(if applicable)* |  |  |  |
| Resources requirements |  |  |  |
| Co-interventions |  |  |  |
| Duration of follow-up |  |  |  |

3. Comparator:

| **Control/comparison** |
| --- |
| □ no intervention □ other (specify:_____________________________) |

4. Outcomes:

| **Primary Outcome: Quality of the Review (Manuscript or peer review report)** | | | | | | |
| --- | --- | --- | --- | --- | --- | --- |
| □ RQI □ other (specify:_____________________________) | | | | | | |
| □ population-level measure □ individual-level measure | | | | | | |
| Primary outcome of the study? □ yes □ no | | | | | | |
| Time points measured | |  | | | | |
| Time points reported | |  | | | | |
| Unit of measurement | |  | | | | |
| □ Continuous □ Categorical (unordered) □ Categorical (ordered) □ Binary | | | | | | |
| Mean |  | | Standard deviation |  | Standard mean difference |  |
| **Primary Outcome: Rejection rate** | | | | | | |
| □ population-level measure □ individual-level measure | | | | | | |
| Primary outcome of the study? □ yes □ no | | | | | | |
| Time points measured | |  | | | | |
| Time points reported | |  | | | | |
| Unit of measurement | |  | | | | |
| □ Continuous □ Categorical (unordered) □ Categorical (ordered) □ Binary | | | | | | |
| Mean |  | | Standard deviation |  | Standard mean difference |  |
| **Primary Outcome: Time spent on the peer review** | | | | | | |
| □ population-level measure □ individual-level measure | | | | | | |
| Primary outcome of the study? □ yes □ no | | | | | | |
| Time points measured | |  | | | | |
| Time points reported | |  | | | | |
| Unit of measurement | |  | | | | |
| □ Continuous □ Categorical (unordered) □ Categorical (ordered) □ Binary | | | | | | |
| Mean |  | | Standard deviation |  | Standard mean difference |  |
| **Other Outcomes** | | | | | | |
| Specify: | | | | | | |
| □ population-level measure □ individual-level measure | | | | | | |
| Primary outcome of the study? □ yes □ no | | | | | | |
| Time points measured | |  | | | | |
| Time points reported | |  | | | | |
| Unit of measurement | |  | | | | |
| □ Continuous □ Categorical (unordered) □ Categorical (ordered) □ Binary | | | | | | |
| Mean |  | | Standard deviation |  | Standard mean difference |  |

5. Risk of Bias

| **Selection** | |
| --- | --- |
| Random sequence generation | □ high risk □ low risk □ unclear |
| Allocation concealment | □ high risk □ low risk □ unclear |
| **Performance** | |
| Who is blinded? | □ participants □ researchers □ outcome assessors □ statisticians |
| Method for blinding participants and research team |  |
| Blinding | □ high risk □ low risk □ unclear |
| **Detection** | |
| Primary outcome measure | □ objective □ subjective |
| Method for blinding primary outcome assessors |  |
| *Primary outcome* | □ high risk □ low risk □ unclear |
| Secondary outcome measure | □ objective □ subjective |
| Method for blinding secondary outcome assessors |  |
| *Secondary outcome* | □ high risk □ low risk □ unclear |
| Other outcome measure | □ objective □ subjective |
| Method for blinding other outcome assessors |  |
| *Other outcome* | □ high risk □ low risk □ unclear |
| **Attrition** | |
| Percent/number missing data in control group |  |
| Reason(s) for missing data in control group |  |
| Percent/number missing data in intervention arm 1 |  |
| Reason(s) for missing data in intervention arm 1 |  |
| Percent/number missing data in intervention arm 2 |  |
| Reason(s) for missing data in intervention arm 2 |  |
| Appropriate analysis of missing data? | □ yes □ no □ unclear |
| Attrition bias | □ high risk □ low risk □ unclear |
| **Reporting** | |
| Reported primary outcome same as protocol (or methods)? | □ yes □ no □ unclear |
| Reporting bias | □ high risk □ low risk □ unclear |
