# Supplementary material for: Impact of interventions to improve the quality of peer review of biomedical journals: a systematic review and meta-analysis
Source: BMC Med. 2016 Jun 10;14:85. doi: 10.1186/s12916-016-0631-5 (PMC4902984; doi:10.1186/s12916-016-0631-5)
Supplement: Additional file 2: Appendix 2. — General characteristics of the RCTs selected. (DOC 94 kb) [file 12916_2016_631_MOESM2_ESM.doc]

Appendix 2. General characteristics of the RCTs selected

| **Author, Year** | **Intervention** |
| --- | --- |
| **Training/mentoring/feedback** | |
| Callaham, JAMA 2002a  Study 1 | - Intervention group: written feedback by editors, ie, peer reviewers received   - Other peer reviewers’ peer-review reports and the editor’s decision letter (usual process)   - a brief summary of the goals for a high-quality peer review   - the editor’s rating of their peer-review report (feedback) - Comparator group: peer reviewers received   - other peer reviewers’ peer-review reports and the editor’s decision letter (usual process) |
| Callaham, JAMA 2002a  Study 2 | - Intervention group: written feedback by editors, ie, peer reviewers received   - other peer reviewers’ peer-review reports and the editor’s decision letter (usual process)   - a brief summary of the goals for a high-quality peer review   - the editor’s ratings of their peer-review report   - the editor’s ratings of the other peer reviewers’ reports   - a copy of an excellent peer-review report of another manuscript - Comparator group: peer reviewers received   - Other peer reviewers’ peer-review reports and the editor’s decision letter |
| Callaham, 2002 Ann Emerg Med  Study 2 | - Intervention group: a 4-hour interactive workshop on peer review run by 2 senior editors, consisting of presentations and discussion about peer review - Comparator group: peer reviewers did not participate in the workshop |
| Houry, 2012  BMC Med Educ | - Intervention group: standard journal practices + mentoring. Mentees were asked to discuss their peer review with their mentor by email or telephone. Mentors were asked to give feedback about how well the mentees addressed the key elements of a good peer review, what they might have done differently, and how they would rate their own peer review using the editors’ 1- to 5-point quality rating scale.   Mentors were senior peer reviewers (ie, top 50 peer reviewers for at least 2 of the past 4 years) who agreed to be a mentor   - Comparator group: standard journal practices, ie, the new peer reviewers received a packet of information materials and were encouraged to complete an online peer-review training module developed by the journal |
| Schroter, 2004  BMJ | - Intervention group 1: face-to-face training - a full-day face-to-face training session on critical appraisal of clinical trials - written instructions - CD rom with techniques on critical appraisal of RCTs - Intervention group 2: self-training - a self-training package consisting of the same or similar materials used in the workshop intervention. - Comparator group: peer reviewers did not have any training about peer-review   We combined the 2 interventions groups |
| **Statistical peer review** | |
| Arnau, 2003  Med Clin (Barc) | - Intervention group: addition of a statistical peer reviewer - Comparator group: usual process |
| Cobo, 2007  PLOS One | 2*2 factorial design   - Intervention group 1: manuscripts assessed by 2 clinical peer reviewers chosen among the usual pool of peer reviewers used by the journal plus an additional statistical peer reviewer selected among the journal referee pool of 39 experts - Intervention group 2: manuscripts assessed by 2 clinical peer reviewers chosen among the usual pool of peer reviewers used by the journal that received a standard letter providing the reporting guidelines relevant to the study - Intervention group 3: manuscripts assessed by 2 clinical peer reviewers chosen among the usual pool of peer reviewers used by the journal that received a standard letter providing the reporting guidelines relevant to the study (ie, CONSORT, QUOROM, STARD) plus an additional statistical peer reviewer - Comparator group: manuscripts assessed by 2 clinical peer reviewers chosen among the usual pool of peer reviewers used by the journal   We selected intervention group 1 and control group for this analysis |
| **Checklist** | |
| Cobo, 2007  PLOS One | *See above*  We selected intervention group 2 and comparator group for this analysis |
| Cobo, 2011  BMJ | - Intervention group: the usual process plus an additional peer review by a statistical peer reviewer who provided suggestions for adherence to appropriate checklists depending on the topic, such as STROBE, CONSORT, TREND, and looked for missing items from reporting guidelines. - Comparator group: the usual process that includes 2 peer reviewers: either 2 clinical peer reviewers, or 1 clinical peer reviewer and 1 statistical or epidemiological peer reviewer. |
| **Open peer review (ie, identity of peer reviewers revealed to the authors, other peer reviewers, and/or the general public)** | |
| Das Sinha, 1999  Natl Med J India | - Intervention group: peer reviewers were informed that their comments would be exchanged with other reviewers of the same manuscript - Comparator group: peer reviewers received the usual cover letter along with the manuscript |
| Godlee, 1998  JAMA | - Intervention group 1: peer reviewers received a blinded manuscript (authors’ details removed) and were asked to sign their peer-review report - Intervention group 2: peer reviewers received a blinded manuscript and were not asked to sign their report - Intervention group 3: peer reviewers received a unblinded manuscript and were asked to sign their report - Intervention group 4: peer reviewers received a unblinded manuscript and were not were asked to sign their report - Group 5: peer reviewers were not informed of their participation in the study. They received an unblinded manuscript and were asked to sign their report. This group was not considered in the analysis because data were not available.   In the analysis we pooled the groups as follows:   - Groups 1 and 3: open peer reviews - Groups 2 and 4: anonymous   Group 5 was not considered in the analysis |
| Van Rooyen, 1998  JAMA | - Intervention group 1: Open peer review: A pair of peer reviewers received the same manuscript. They were asked to consent to their identity being revealed to their co-peer reviewers. The pair of peer reviewers was randomly allocated to a blinded (removing author identity details from title and acknowledgements)/unblinded version of the manuscript. - Intervention group 2: “the masked group”. A pair of peer reviewers received the same manuscript. They were not asked to consent to their identity being revealed. The pair of peer reviewers was randomly allocated to a blinded/unblinded version of the manuscript. - Uninformed group: The peer reviewers were not informed of the study   We selected intervention group 1 and 2 for this analysis. The uninformed group was not considered in the analysis |
| Van Rooyen, 1999  BMJ | - Intervention group: peer reviewers informed that their identity would be revealed to authors - Comparator group: peer reviewers remained anonymous to authors |
| Van Rooyen, 2010  BMJ | - Intervention group: peer reviewers informed that their review would be posted online - Comparator group: peer reviewers informed that their identity would be revealed only to authors |
| Vinther, 2012  Dan Med | - Intervention group: peer reviewers were asked to have their identity revealed to authors - Comparator group: peer reviewers remained anonymous to authors |
| Walsh, 2000  Br J Psychiatry | - Intervention group: peer reviewers' identities revealed to the authors - Comparator group: peer reviewers remained anonymous as per usual procedures |
| **Blinded peer review (ie, peer reviewers blinded to the authors’ names and affiliations)** | |
| Alam, 2011  Br J Dermatol | For each manuscript included:   - Intervention group: blinded version of the manuscript (ie, authors’ name, affiliations and bibliography/endnotes section removed) - Comparator group: unblinded version of the manuscript (ie, with authors’ name, affiliations and bibliography/endnotes section ) |
| Fisher, 1994  JAMA | For each manuscript included:   - Intervention group: blinded version of the manuscript (ie, authors’ name removed) - Comparator group: unblinded version of the manuscript (ie, with authors’ name) |
| Godlee, 1998  JAMA | - Intervention group 1: peer reviewers received a blinded manuscript (authors’ details removed) and were asked to sign their peer-review report - Intervention group 2: peer reviewers received a blinded manuscript and were not asked to sign their report - Intervention group 3: peer reviewers received a unblinded manuscript and were asked to sign their report - Intervention group 4: peer reviewers received a unblinded manuscript and were not were asked to sign their report - Group 5: peer reviewers were not informed of their participation in the study. They received an unblinded manuscript and were asked to sign their report. This group was not considered in the analysis because data were not available.   In the analysis we pooled the groups as follows:   - Groups 1 and 2: open peer reviews - Groups 3 and 4: anonymous   Group 5 was not considered in the analysis |
| Justice, 1998  JAMA | - Intervention group: blinded version of the manuscript (ie, authors’ name and institutional identity removed) - Comparator group: unblinded version of the manuscript (ie, with authors’ name and institutional identity) |
| McNutt, 1990  JAMA | For each manuscript randomized:  Intervention group: blinded version of the manuscript (ie, authors’ name and institutional identity removed)   - Comparator group: unblinded version of the manuscript (ie, with authors’ name and institutional identity) |
| Van Rooyen, 1998  JAMA | Intervention group: blinded version of the manuscript submitted to reviewers. (i.e authors name and institutional identity removed)   - Comparator group: unblinded version of the manuscript submitted to reviewers   This study also assessed masked versus unmasked review (See above in open peer-review intervention) |
| **Accelerate the peer-review process** | |
| Johnston, 2007  Ann Neurol | - Intervention group: manuscripts were initially evaluated by editors who decided if they would send it to peer reviewers - Comparator group: systematic formal external review by outside experts |
| Neuhauser, 1989  Medical Care | - Intervention group: editors called potential reviewers before sending them a manuscript to review - Comparator group: editors sent out manuscripts for review without a prior call |
| Pitkin, 2002  JAMA | - Intervention group: the “ask-first” group. Referees received by fax information about the manuscript and were asked to indicate their willingness by return fax. If they refused or failed to respond within 3 working days, a substitute chosen by the editor was contacted by fax and so on until a referee agreed to the review. - Comparator group: the “just-send” group. Referees assigned were mailed manuscripts and asked to return their reviews by fax or e-mail within 3 weeks; if unable to comply, they were to telephone the editorial office immediately, and a substitute was chosen. |
