# Supplementary material for: Impact of interventions to improve the quality of peer review of biomedical journals: a systematic review and meta-analysis
Source: BMC Med. 2016 Jun 10;14:85. doi: 10.1186/s12916-016-0631-5 (PMC4902984; doi:10.1186/s12916-016-0631-5)
Supplement: Additional file 3: Appendix 3. — Risk of bias summary: the review authors’ judgments about each risk of bias item for each included study. (DOC 278 kb) [file 12916_2016_631_MOESM3_ESM.doc]

Appendix 3. Risk of Bias Summary: the review authors’ judgments about each risk of bias item for each included study

**Training/Mentoring/Feedback**

**Callaham, JAMA 2002**

**Study 1**

**Outcome:** the quality of peer review report

|  |  |  |
| --- | --- | --- |
| **Bias** | **Authors' judgment** | **Support for judgment** |
| *Random sequence generation (selection bias)* | **Low risk** | Computer-generated randomization sequence. (StatView 5.0, version 5.02, SAS) |
| *Allocation concealment (selection bias)* | **Low risk** | Reported by authors: “Therefore all allocation was by computer algorithm.” |
| *Blinding of outcome assessment (detection bias)* | **Low risk** | Reported by authors: "All editors were blinded to study purpose and reviewer participation." |
| *Incomplete outcome data (attrition bias)* | **High risk** | Very low response rate of eligible subjects for both RCTs included in this paper. "Fifty-one reviewers were eligible for entry, but 16 (10 control subjects and 6 intervention subjects) completed insufficient rate reviews during the study period, leaving 35 with sufficient data for analysis who completed 182 reviews.” |

**Callaham, JAMA 2002**

**Study 2**

**Outcome:** the quality of peer review report

|  |  |  |
| --- | --- | --- |
| **Bias** | **Authors' judgment** | **Support for judgment** |
| *Random sequence generation (selection bias)* | **Low risk** | Computer-generated randomization sequence. (StatView 5.0, version 5.02, SAS) |
| *Allocation concealment (selection bias)* | **Low risk** | Reported by authors: “Therefore all allocation was by computer algorithm.” |
| *Blinding of outcome assessment (detection bias)* | **Low risk** | Reported by authors: "All editors were blinded to study purpose and reviewer participation." |
| *Incomplete outcome data (attrition bias)* | **High risk** | Very low response rate of eligible subjects for both RCTs included in this paper. Study 2: "One hundred twenty-seven reviewers were eligible; 32 (15 control subjects and 17 intervention subjects) did not complete sufficient reviews, leaving 95 with sufficient data for analysis (324 reviews)." |

**Callaham, Ann Emerg Med 2002**

**Study 2**

**Outcome:** the quality of peer-review report

|  |  |  |
| --- | --- | --- |
| **Bias** | **Authors' judgment** | **Support for judgment** |
| *Random sequence generation (selection bias)* | **Low risk** | Computer-generated randomization sequence. (StatView 5.0, SAS) |
| *Allocation concealment (selection bias)* | **Low risk** | Reported by authors: “Therefore all allocation was by computer algorithm.” |
| *Blinding of outcome assessment (detection bias)* | **Low risk** | Reported by authors: "Editors were blinded as to the identity of study participants, whether they had attended the workshop, and the purpose of both studies." |
| *Incomplete outcome data (attrition bias)* | **High risk** | High rate of follow-up |

**Houry, 2012**

**Outcome:** the quality of peer-review report

|  |  |  |
| --- | --- | --- |
| **Bias** | **Authors' judgment** | **Support for judgment** |
| *Random sequence generation (selection bias)* | **Low risk** | "We used computer-generated randomization to assign new reviewers to either the intervention or control group." |
| *Allocation concealment (selection bias)* | **Unclear risk** | Insufficient information to permit judgment of Yes or No. The method of concealment was not described or was not described in sufficient detail to allow a definite judgment. |
| *Blinding of outcome assessment (detection bias)* | **Unclear risk** | "Over the course of the study, editors invited reviewers in their standard fashion, without the knowledge of which new reviewers were assigned to the mentorship or control groups." It is unclear whether or not editors were not blinded at the time of outcome assessment. |
| *Incomplete outcome data (attrition bias)* | **Low risk** | 4 participants excluded from the analysis in the control group |

**Schroter, 2004**

|  |  | |  |
| --- | --- | --- | --- |
| **Bias** | **Authors' judgment** | | **Support for judgment** |
| *Random sequence generation (selection bias)* | **Low risk** | | Reported by authors: "We randomised consenting reviewers into three groups: two intervention groups and a control group. We used a stratified permuted blocks randomisation method to ensure that the groups were similar in terms of factors known to influence the quality of reviews (age, current investigators in medical research projects, postgraduate training in epidemiology, postgraduate training in statistics, and editorial board members of a scientific or medical journal)." |
| *Allocation concealment (selection bias)* | **Low risk** | | Reported by authors: “Authors sent the statistician an anonymised list of study ID numbers for reviewers who agreed to take part in the study along with key the variables (age, current investigators in medical research projects, postgraduate training in epidemiology, postgraduate training in statistics, and editorial board members of a scientific or medical journal)  so he could do the stratified permuted blocks randomization.” |
| *Blinding of outcome assessment (detection bias)* | **Time spent** | **Low risk** | Reported by authors: Peer reviewers were aware of their participation in the trial, but they were not aware of the study hypothesis. |
|  | **Rejection rate** | **Low risk** | Reported by authors: Peer reviewers were aware of their participation in the trial, but they were not aware of the study hypothesis. |
|  | **Quality of**  **peer review**  **report** | **Low risk** | This was assessed by 2 editors who were blinded to the identity and group allocation of the reviewers. |
| *Incomplete outcome data (attrition bias)* | **Time spent** | **High risk** | "As the difference between responders and non-responders is unknown, the impact of non-response on the conclusions cannot be definitively determined." |
|  | **Rejection rate** | **High risk** |  |
|  | **Quality of**  **peer review**  **report** | **High risk** |  |

**Statistical peer review**

**Arnau 2003**

**Outcome:** the final manuscript quality

|  |  |  |
| --- | --- | --- |
| **Bias** | **Authors' judgment** | **Support for judgment** |
| ***Random sequence generation (selection bias)*** | **Low risk** | Computer-generated randomization sequence. |
|  |  | Translation: The randomization list, in blocks of 4, was generated computationally by an external statistician and was filed in the offices of "Clinical Medicine". The data entry and validation was performed at the Polytechnic University of Catalonia (UPC). |
| ***Allocation concealment (selection bias)*** | **Low risk** | Reported by authors: “The methodological editor previously generated the random 1 to 1 allocation list with blocks of 4 (this restriction was not known by the remaining investigators).” |
| ***Blinding of outcome assessment (detection bias)*** | **Low risk** | From English abstract: "Two evaluators, blinded to the paper's group, assessed the quality improvement in both groups, from submission to publication using a modified version of the Goodman et al. scale." Blinding of outcome assessment, and unlikely that the blinding could have been broken. |
| ***Incomplete outcome data (attrition bias)*** | **High risk** | 82 manuscripts randomized (41 in each group), but only 43 analyzed (21 in intervention group and 22 in control group). In the intervention group the reasons for exclusion were administrative error, arrival back after the deadline, no response of statistician or no sending the revision version. In the control group, the reasons for exclusion were administrative error and arrival back after the deadline. |

**Cobo, 2007**

**Outcome:** the final manuscript quality

|  |  |  |
| --- | --- | --- |
| **Bias** | **Authors' judgment** | **Support for judgment** |
| *Random sequence generation (selection bias)* | **Low risk** | "Then, manuscripts were randomly allocated (by AS) using a computer program that first stratifies by study type, and second allocates to intervention groups while minimizing differences in initial quality." |
| *Allocation concealment (selection bias)* | **Low risk** | Reported by authors: “As it was obtained by a minimization algorithm, the allocated group was generated after the paper inclusion as follows: 1) two clinical editors (FC and JMR) included consecutively in the study all manuscripts potentially suitable for publication; 2) those editors assigned the standard clinical reviewers; 3) the methodological editor (EC) obtained the baseline data needed for the minimization algorithm; 4) a third clinical editor (AS) obtained the paper allocation through the minimization algorithm; and 5) the two crossed interventions were added to the corresponding groups: (a) the methodological editor added the statistical reviewer; and (b) an editorial assistant added the standard reporting guideline  suggestion.” |
| *Blinding of outcome assessment (detection bias)* | **Unclear risk** | "Two evaluators independently rated the reporting quality of manuscripts at initial submission and following peer review and revision, according to the MQAI. Both knew the initial and final status but were blinded to the intervention group."  “On the ‘presence of a statistical reviewer’ question, 20 (20.2%) originals were evaluated as ‘I don’t know.’ The remaining 79 originals (79.8%) were inspected with a match percentage of 60.8% (95% CI 49.1 to 71.6%).” |
| *Incomplete outcome data (attrition bias)* | **Low risk** | 129 manuscripts were randomized: 37 in the group clinical reviewers, 31 in the group clinical + statistician reviewers, 32 in the group clinical + checklist reviewer and 29 in the group clinical + statistician + checklist. 115 manuscripts analyzed: 32 in the group clinical reviewers, 30 in the group clinical + statistician reviewers, in the group 28 clinical + checklist reviewer and in the group 25 clinical + statistician + checklist. Several sensitivity analyses were performed and produced very similar conclusions. |

**Checklist**

**Cobo, 2007**

**Outcome:** the final manuscript quality

|  |  |  |
| --- | --- | --- |
| **Bias** | **Authors' judgment** | **Support for judgment** |
| *Random sequence generation (selection bias)* | **Low risk** | "Then, manuscripts were randomly allocated (by AS) using a computer program that first stratifies by study type, and second allocates to intervention groups while minimizing differences in initial quality." |
| *Allocation concealment (selection bias)* | **Low risk** | Reported by authors: “As it was obtained by a minimization algorithm, the allocated group was generated after the paper inclusion as follows: 1) two clinical editors (FC and JMR) included consecutively in the study all manuscripts potentially suitable for publication; 2) those editors assigned the standard clinical reviewers; 3) the methodological editor (EC) obtained the baseline data needed for the minimization algorithm; 4) a third clinical editor (AS) obtained the paper allocation through the minimization algorithm; and 5) the two crossed interventions were added to the corresponding groups: (a) the methodological editor added the statistical reviewer; and (b) an editorial assistant added the standard reporting guideline  suggestion.” |
| *Blinding of outcome assessment (detection bias)* | **Unclear risk** | Reported by authors: "Two evaluators independently rated the reporting quality of manuscripts at initial submission and following peer review and revision, according to the MQAI. Both knew the initial and final status but were blinded to the intervention group."  “On the use of checklist, the evaluators were able to guess the intervention group in 65.3% (95% CI 53.5% to 76%) of the 75/99 (75.8%) cases analyzed.” |
| *Incomplete outcome data (attrition bias)* | **Low risk** | 129 manuscripts were randomized: 37 in the group clinical reviewers, 31 in the group clinical + statistician reviewers, 32 in the group clinical + checklist reviewer and 29 in the group clinical + statistician + checklist. However, 115 manuscripts were analyzed: 32 in the group clinical reviewers, 30 in the group clinical + statistician reviewers, in the group 28 clinical + checklist reviewer and in the group 25 clinical + statistician + checklist. Several sensitivity analyses were performed and produced very similar conclusions. |

**Cobo, 2011**

**Outcome: the final manuscript quality**

|  |  |  |
| --- | --- | --- |
| **Bias** | **Authors' judgment** | **Support for judgment** |
| *Random sequence generation (selection bias)* | **Low risk** | "Before randomisation, all manuscripts were given an ad hoc assessment by the senior statistician (EC) using a score ranging from 1 to 9, in order to give a global measure of report quality at baseline. With these scores, we were able to use a random minimisation algorithm to balance mean differences in the ad hoc score as well as differences in study type counts (that is, intervention, longitudinal, cross sectional and other type), but not to equilibrate the overall number of manuscripts in both groups. The algorithm gave probabilities from 0.5 (in the case of indifferent allocation to one or another group) to 0.8 (if both minimisation factors indicated allocation to the same group)." |
| *Allocation concealment (selection bias)* | **Low risk** | "The second editorial decision (after peer review and before randomisation) took place without committee members knowing which papers were allocated to receive the additional review (fig 1). At later editorial decisions, committee members was the additional reviews of papers in the intervention group." |
| *Blinding of outcome assessment (detection bias)* | **Unclear risk** | Reported by authors: “The three raters (recently graduates in statistics) were truly masked to the group and they didn't have access to the reviewer comments during their evaluation (first study phase). But they commented that some modifications in the new reviewed version of the paper included sentences that (they though that) they recognized either from myself or as standard RG phrases. For this reason, authors asked them if they recognized or not the group. In applying the majority rule, the joint opinion of the 3 raters guessed the intervention group in 62% of the papers (95% CI 51% to 72%), excluding the 50% of expected guesses (if random)." |
| *Incomplete outcome data (attrition bias)* | **Low risk** | 92 manuscripts randomized (41 in the control group and 51 in the control group) and 92 manuscripts analyzed. |

**Open peer review**

**Das Sinha, 1999**

|  |  | |  |
| --- | --- | --- | --- |
| **Bias** | **Authors' judgment** | | **Support for judgment** |
| *Random sequence generation (selection bias)* | **Low risk** | | Reported by authors: "Randomization was done using random number charts and the sealed envelope technique." |
| *Allocation concealment (selection bias)* | **Low risk** | | Reported by authors: "Randomization was done using random number charts and the sealed envelope technique." |
| *Blinding of outcome assessment (detection bias)* | **Rejection rate** | **Low risk** | The further aim was to evaluate whether lack of blinding affected their decision. |
|  | **Quality of**  **peer review**  **report** | **Low risk** | "The two editors were unaware of the reviewers' names, nationality, institutional affiliations, and whether or not they had been told that their comments would be exchanged." |
| *Incomplete outcome data (attrition bias)* | **Rejection rate** | **Low risk** | No mention of differences between groups in attrition reasons, nor any methods to estimate and account for missing data. Authors did not give the standard deviation for the time taken by groups. |
|  | **Quality of**  **peer review**  **report** | **Low risk** |  |

**Godlee, 1998**

**Outcome:** The rejection rate

|  |  |  |
| --- | --- | --- |
| **Bias** | **Authors' judgment** | **Support for judgment** |
| *Random sequence generation (selection bias)* | **Low risk** | "A statistician gave each reviewer a random number. These 670 reviewers were ordered and the first 420 were selected for allocation to 5 groups in random number sequence. Four of these groups were constructed, using factorial design, to investigate the effects of blinding reviewers to the authors' identities and asking them to sign their reports." |
| *Allocation concealment (selection bias)* | **Low risk** | The person who performed randomization was independent of the review. |
| *Blinding of outcome assessment (detection bias)* | **Low risk** | "Each reviewer's report was assessed independently by an editor and an epidemiologist, neither of whom was aware of the group to which the reviewer had been allocated. Where there was disagreement, the report was re-examined and consensus was reached." |
| *Incomplete outcome data (attrition bias)* | **High risk** | "Among the 90 respondents who had been blinded to the authors' identities, 23 (26%) named the authors correctly in their report. Rate ratios were little changed when those people were excluded." |

V**an Rooyen, 1998**

| **Bias** | **Authors' judgment** | | **Support for judgment** |
| --- | --- | --- | --- |
| *Random sequence generation (selection bias)* | **Low risk** | | "The randomization process was undertaken by a researcher who was independent from the editorial decision-making process using a computerized minimization program with a random component." |
| *Allocation concealment (selection bias)* | **Unclear risk** | | Insufficient information to permit judgment of Yes or No. The method of concealment was not described or was not described in sufficient detail to allow a definite judgment. |
| *Blinding of outcome assessment (detection bias)* | **Time spent** | **Low risk** | Reported by authors: “Peer reviewers were aware of their participation in the trial but they were not aware of the study hypothesis.” |
|  | **Quality of**  **peer review**  **report** | **Low risk** | "At no stage were editors or authors aware of the group to which a manuscript [was] allocated." |
| *Incomplete outcome data (attrition bias)* | **Time spent** | **Unclear**  **risk** | “Of the 527 manuscripts entered into the study, 487 (89%) were successfully randomized and followed up.” |
|  | **Quality of**  **peer review**  **report** | **Unclear**  **risk** |  |

V**an Rooyen, 1999**

| **Bias** | **Authors' judgment** | | **Support for judgment** |
| --- | --- | --- | --- |
| *Random sequence generation (selection bias)* | **Low risk** | | "Randomisation was carried out by a researcher using a computerised randomisation program." |
| *Allocation concealment (selection bias)* | **Unclear risk** | | Insufficient information to permit judgment of Yes or No. The method of concealment was not described or was not described in sufficient detail to allow a definite judgment. |
| *Blinding of outcome assessment (detection bias)* | **Time**  **spent** | **Low risk** | Reported by authors: “Peer reviewers were aware of their participation in the trial but they were not aware of the study hypothesis.” |
|  | **Rejection rate** | **Low risk** | The further aim was to evaluate whether lack of blinding affected their decision. |
|  | **Quality of**  **Peer review**  **report** | **Low risk** | "Editors did not know which of the reviewers had consented to be identified to the author." |
| *Incomplete outcome data (attrition bias)* | **Time**  **spent** | **High risk** | Of the 250 reviewers initially invited to participate, 73 declined: 29 (23%) in the group randomised to remain anonymous (anonymous reviewers) and 44 (35%) in the group randomised to be asked for consent to be identified (identified reviewers). For 11 of the 125 randomised papers, both reviewers declined, in only 18 the anonymous reviewer declined, and in only 33 the identified reviewer declined. Thus, the difference between identified reviewers and anonymous reviewers in declining to review was 12% (35% vs 23%, 95% CI 0.2% to 24%), which is marginally significant (McNemar's χ2= 3.84, P = 0.0499). |
|  | **Rejection rate** | **High risk** |  |
|  | **Quality of**  **Peer review**  **report** | **High risk** |  |

**Van Rooyen, 2010**

|  |  | |  |
| --- | --- | --- | --- |
| **Bias** | **Authors' judgment** | | **Support for judgment** |
| *Random sequence generation (selection bias)* | **Unclear risk** | | No information gave by authors about the random sequence generation. |
| *Allocation concealment (selection bias)* | **Unclear risk** | | Insufficient information to permit judgment of Yes or No. The method of concealment was not described or was not described in sufficient detail to allow a definite judgment. |
| *Blinding of outcome assessment (detection bias)* | **Time spent** | **Low risk** | Peer reviewers did not seem to be aware of the study hypothesis. |
|  | **Rejection rate** | **Low risk** | The further aim was to evaluate whether lack of blinding affected their decision. |
|  | **Quality of**  **peer review**  **report** | **Low risk** | "Editors were blinded throughout to the group allocation." |
| *Incomplete outcome data (attrition bias)* | **Time spent** | **Low risk** | 225/265 (85%) randomized manuscripts were analyzed in the intervention group and 246/283 (87%) in the control group. |
|  | **Rejection rate** | **Low risk** |  |
|  | **Quality of**  **peer review**  **report** | **Low risk** |  |

**Vinther, 2012**

|  |  | |  |
| --- | --- | --- | --- |
| **Bias** | **Authors' judgment** | | **Support for judgment** |
| *Random sequence generation (selection bias)* | **Unclear risk** | | Insufficient information about the sequence generation process to permit judgment of Yes or No. |
| *Allocation concealment (selection bias)* | **Unclear risk** | | Insufficient information to permit judgment of Yes or No. The method of concealment was not described or was not described in sufficient detail to allow a definite judgment. |
| *Blinding of outcome assessment (detection bias)* | **Rejection rate** | **Low risk** | The further aim was to evaluate whether lack of blinding affected their decision. |
|  | **Quality of**  **Peer review**  **report** | **Low risk** | "Review quality was assessed by two editors, neither of whom was aware of the group to which a reviewer had been allocated." |
| *Incomplete outcome data (attrition bias)* | **Rejection rate** | **Low risk** | "For eight manuscripts, it was not possible to obtain complete data (an open and a blinded review) despite that several reminders were sent to reviewers; these eight manuscripts (corresponding to 1 reviews) were excluded from the analysis." Missing data represented less than 5% in the intervention and control groups. |
|  | **Quality of**  **Peer review**  **report** | **Low risk** |  |

**Walsh, 2000**

|  |  | |  |
| --- | --- | --- | --- |
| **Bias** | **Authors' judgment** | | **Support for judgment** |
| *Random sequence generation (selection bias)* | **Low risk** | | "Simple randomization was performed using computer-generated random numbers." |
| *Allocation concealment (selection bias)* | **Unclear risk** | | Insufficient information to permit judgment of Yes or No. The method of concealment was not described or was not described in sufficient detail to allow a definite judgment. |
| *Blinding of outcome assessment (detection bias)* | **Time spent** | **Low risk** | Peer reviewers did not seem to be aware of the study hypothesis. |
|  | **Rejection rate** | **Low risk** | The further aim was to evaluate whether lack of blinding affected their decision. |
|  | **Quality of**  **peer review**  **report** | **Low risk** | "Throughout the trial period, reviews returned by those who had declined to reveal their names to authors were rated on quality. These reviews were rated blind, interspersed with the other reviews, by the Trainee Editors." "...use of blind ratings and good inter-rater reliability as measured by weighted k statistics." |
| *Incomplete outcome data (attrition bias)* | **Time spent** | **High risk** | "It was thought that the 'decliners' group may differ systematically from the participating group in terms of review quality. An attempt was made to estimate any differences." 57 refused to participate because they did not want to be in the "open" review group. Otherwise, those who were not followed up (did not complete the review) was approximately the same in the control and "open" review groups (12% and 13% respectively). |
|  | **Rejection rate** | **High risk** |  |
|  | **Quality of**  **peer review**  **report** | **High risk** |  |

**Blinded peer review**

**Alam, 2011**

**Outcome: The rejection rate**

|  |  |  |
| --- | --- | --- |
| **Bias** | **Authors' judgment** | **Support for judgment** |
| *Random sequence generation (selection bias)* | **Unclear risk** | Insufficient information about the sequence generation process to permit judgment of Yes or No. |
| *Allocation concealment (selection bias)* | **Unclear risk** | Insufficient information to permit judgment of Yes or No. The method of concealment was not described or was not described in sufficient detail to allow a definite judgment. |
| *Blinding of outcome assessment (detection bias)* | **Low risk** | The further aim was to evaluate whether lack of blinding affected their decision. |
| *Incomplete outcome data (attrition bias)* | **Low risk** | "In a total of six cases, reviewers failed to complete their reviews within 1 month of the date of assignment; a different reviewer was then randomly assigned to replace each of these. Replacement reviewers were substituted for three blinded and three unblinded reviewers, and in no instance were multiple replacement reviewers required for a given manuscript.” |

**Fisher, 1994**

**Outcome:** The rejection rate

|  |  |  |
| --- | --- | --- |
| **Bias** | **Authors' judgment** | **Support for judgment** |
| *Random sequence generation (selection bias)* | **Low risk** | "Which reviewers were blinded was determined by use of a computer-generated random-numbers table." |
| *Allocation concealment (selection bias)* | **Unclear risk** | Insufficient information to permit judgment of Yes or No. The method of concealment was not described or was not described in sufficient detail to allow a definite judgment. |
| *Blinding of outcome assessment (detection bias)* | **Low risk** | The further aim was to evaluate whether lack of blinding affected their decision. |
| *Incomplete outcome data (attrition bias)* | **Low risk** | "Two blinded and six nonblinded reviewers did not return their reviews."  Concerning the rejection rate, the worst case study showed an impact of missing data. |

**Godlee, 1998**

**Outcome:** The rejection rate

|  |  |  |
| --- | --- | --- |
| **Bias** | **Authors' judgment** | **Support for judgment** |
| *Random sequence generation (selection bias)* | **Low risk** | "A statistician gave each reviewer a random number. These 670 reviewers were ordered and the first 420 were selected for allocation to 5 groups in random number sequence. Four of these groups were constructed, using factorial design, to investigate the effects of blinding reviewers to the authors' identities and asking them to sign their reports." |
| *Allocation concealment (selection bias)* | **Low risk** | The person who performed randomization was independent of the review. |
| *Blinding of outcome assessment (detection bias)* | **Low risk** | "Each reviewer's report was assessed independently by an editor and an epidemiologist, neither of whom was aware of the group to which the reviewer had been allocated. Where there was disagreement, the report was re-examined and consensus was reached." |
| *Incomplete outcome data (attrition bias)* | **High risk** | "Among the 90 respondents who had been blinded to the authors' identities, 23 (26%) named the authors correctly in their report. Rate ratios were little changed when those people were excluded." |

**Justice, 1998**

**Outcome:** the quality of peer review report

|  |  |  |
| --- | --- | --- |
| **Bias** | **Authors' judgment** | **Support for judgment** |
| *Random sequence generation (selection bias)* | **Low risk** | "Randomization was performed using random number tables." |
| *Allocation concealment (selection bias)* | **Unclear risk** | Insufficient information to permit judgment of Yes or No. The method of concealment was not described or was not described in sufficient detail to allow a definite judgment. |
| *Blinding of outcome assessment (detection bias)* | **Low risk** | "The manuscript editor and the corresponding author rated the quality of each review, unaware of the group to which the review had been assigned." |
| *Incomplete outcome data (attrition bias)* | **Unclear risk** | "Of those randomized to the intervention, 77 (84%) of 92 had sufficient data to compare a review quality based on the editor's judgment." No mention of the missing data in the control group. |

**McNutt, 1990**

**Outcome:** the quality of peer review report

|  |  |  |
| --- | --- | --- |
| **Bias** | **Authors' judgment** | **Support for judgment** |
| *Random sequence generation (selection bias)* | **Low risk** | Sufficient information about the sequence generation process to permit judgment of Yes or No. |
| *Allocation concealment (selection bias)* | **Unclear risk** | Insufficient information to permit judgment of Yes or No. The method of concealment was not described or was not described in sufficient detail to allow a definite judgment. |
| *Blinding of outcome assessment (detection bias)* | **Low risk** | "After reviews were returned, the manuscript and the reviews were given to an editor who was blinded to the authors of the manuscript and their institutions and to the identity of the reviewers. The editor then graded the quality of both the manuscript and its reviews." |
| *Incomplete outcome data (attrition bias)* | **Low risk** | 131 manuscripts randomized, 127 in the intervention group and 125 in control group were analyzed. Missing data represented less than 10% in the intervention and control groups. |

V**an Rooyen, 1998**

| **Bias** | **Authors' judgment** | | **Support for judgment** |
| --- | --- | --- | --- |
| *Random sequence generation (selection bias)* | **Low risk** | | "The randomization process was undertaken by a researcher who was independent from the editorial decision-making process using a computerized minimization program with a random component." |
| *Allocation concealment (selection bias)* | **Unclear risk** | | Insufficient information to permit judgment of Yes or No. The method of concealment was not described or was not described in sufficient detail to allow a definite judgment. |
| *Blinding of outcome assessment (detection bias)* | **Time spent** | **Low risk** | Reported by authors: “Peer reviewers were aware of their participation in the trial but they were not aware of the study hypothesis.” |
|  | **Quality of**  **peer review**  **report** | **Low risk** | "At no stage were editors or authors aware of the group to which a manuscript [had] been allocated." |
| *Incomplete outcome data (attrition bias)* | **Time spent** | **Unclear Risk** | “Of the 527 manuscripts entered into the study, 487 (89%) were successfully randomized and followed up.” |
|  | **Quality of**  **peer review**  **report** | **Unclear risk** |  |

**Accelerate the peer review**

**Johnston, 2007**

**Outcome: accelerate the peer review process**

|  |  |  |
| --- | --- | --- |
| **Bias** | **Authors' judgment** | **Support for judgment** |
| *Random sequence generation (selection bias)* | **Unclear risk** | Insufficient information to permit judgment of Yes or No. The method of concealment was not described or was not described in sufficient detail to allow a definite judgment. |
| *Allocation concealment (selection bias)* | **Low risk** | “Without knowledge of randomization group, one of our six editors made a decision about whether a manuscript should be sent out for review or rejected without further consideration.” |
| *Blinding of outcome assessment (detection bias)* | **Low risk** | The outcome was the delay of the peer review process, so the blinding was not necessary for the assessment. |
| *Incomplete outcome data (attrition bias)* | **Low risk** | All manuscripts included were analyzed. |

**Neuhauser, 1989**

**Outcome: accelerate the peer review process**

|  |  |  |
| --- | --- | --- |
| **Bias** | **Authors' judgment** | **Support for judgment** |
| *Random sequence generation (selection bias)* | **Unclear risk** | Insufficient information to permit judgment of Yes or No. The method of concealment was not described or was not described in sufficient detail to allow a definite judgment. |
| *Allocation concealment (selection bias)* | **Unclear risk** | Insufficient information to permit judgment of Yes or No. The method of concealment was not described or was not described in sufficient detail to allow a definite judgment. |
| *Blinding of outcome assessment (detection bias)* | **Low risk** | The outcome was the delay of the peer review process, so the blinding was not necessary for the assessment. |
| *Incomplete outcome data (attrition bias)* | **Low risk** | “Ninety-five percent of called manuscripts were reviewed compared with 92% of noncalled manuscripts, an insignificant difference.” |

**Pitkin, 2002**

**Outcome: accelerate the peer review process**

|  |  |  |
| --- | --- | --- |
| **Bias** | **Authors' judgment** | **Support for judgment** |
| *Random sequence generation (selection bias)* | **Low risk** | “Using a random-number generator, an editorial assistant assigned one referee to justsend and the other to askfirst.” |
| *Allocation concealment (selection bias)* | **Low risk** | “Using a random-number generator, an editorial assistant assigned one referee to justsend and the other to askfirst.” |
| *Blinding of outcome assessment (detection bias)* | **Low risk** | The outcome was the delay of the peer review process, so the blinding was not necessary for the assessment. |
| *Incomplete outcome data (attrition bias)* | **Low risk** | All manuscripts included were analyzed. |
